# Supplementary material for: Ultrasound-Assisted Extraction of Taxifolin, Diosmin, and Quercetin from Abies nephrolepis (Trautv.) Maxim: Kinetic and Thermodynamic Characteristics
Source: Molecules. 2020 Mar 19;25(6):1401. doi: 10.3390/molecules25061401 (PMC7144359; doi:10.3390/molecules25061401)
Supplement: Supplementary file 1 [file molecules-25-01401-s001.pdf]

Table S1. Box-Behnken design (BBD) with experimental value for total yield of the three flavonoids[a].

RUN	X_1[b]	X_2	X_3	Y_{Leaves}[c]	Taxifolin	Diosmin×100	Quercetin	Y_{bark}	Taxifolin	Diosmin×100	Quercetin
	min	W	K	mg/g	mg/g	mg/g	mg/g	mg/g	mg/g	mg/g	mg/g
1	50	160	313.15	75.37	23.67	21.25	51.49	8.44	1.08	2.59	7.33
2	30	200	323.15	85.82	25.72	22.86	56.87	12.23	1.16	3.72	11.03
3	50	200	323.15	87.23	26.03	21.97	60.98	11.45	1.05	4.32	10.36
4	40	200	313.15	76.35	23.99	21.36	52.15	11.42	1.08	3.48	10.30
5	40	160	323.15	94.21	27.35	25.64	66.60	13.87	1.53	4.29	12.30
6	40	120	313.15	90.29	26.56	24.37	63.49	9.88	1.74	3.87	8.10
7	40	200	333.15	98.12	29.81	26.55	66.05	14.19	1.35	4.32	12.81
8	30	120	323.15	96.34	29.76	26.41	66.32	13.98	1.98	4.20	11.96
9	40	120	333.15	99.56	29.93	26.77	69.36	16.23	2.27	4.57	13.91
10	40	160	323.15	101.45	30.32	27.41	70.86	17.65	2.59	4.74	15.01
11	40	160	323.15	101.45	30.32	27.41	70.86	17.65	2.59	4.74	15.01
12	40	160	323.15	101.45	30.32	27.41	70.86	17.65	2.59	4.74	15.01
13	40	160	323.15	101.45	30.32	27.41	70.86	17.65	2.59	4.74	15.01
14	50	120	323.15	87.32	26.15	21.43	60.96	9.52	1.54	3.75	7.94
15	30	160	333.15	99.37	29.54	26.16	69.57	15.49	1.46	4.53	13.98
16	30	160	313.15	80.34	24.51	21.44	55.62	9.08	1.87	3.29	7.18
17	50	160	333.15	98.32	29.73	37.51	68.21	10.21	1.47	3.48	8.71

[a] The results were obtained with Design Expert 8.0.6 software; [b] X_1 is the extraction time (min); X_2 is the ultrasonic irradiation power (W); X_3 is the temperature (K); [c] Y_{Leaves} is the total yield of 3 flavonoids from leaves of *A. nephrolepis*; Y_{bark} is the total yield of 3 flavonoids from bark of *A. nephrolepis*.
